# SATSN: A Spatial-Adaptive Two-Stream Network for Automatic Detection of Giraffe Daily Behaviors

**DOI:** 10.3390/ani15192833

**Published:** 2025-09-28

**Authors:** Haiming Gan, Xiongwei Wu, Jianlu Chen, Jingling Wang, Yuxin Fang, Yuqing Xue, Tian Jiang, Huanzhen Chen, Peng Zhang, Guixin Dong, Yueju Xue

**Affiliations:** 1 College of Electronic Engineering, South China Agricultural University, Guangzhou 510642, China; haiminggan@scau.edu.cn (H.G.); xiongweiwu@stu.scau.edu.cn (X.W.); lucus@stu.scau.edu.cn (J.C.); 202434210225@stu.scau.edu.cn (J.W.); fangyuxin@stu.scau.edu.cn (Y.F.); 202434210230@stu.scau.edu.cn (Y.X.); chz_@stu.scau.edu.cn (H.C.); 2 College of Mathematics and Informatics, South China Agricultural University, Guangzhou 510642, China; jiangtian@scau.edu.cn; 3 Chimelong Group Co., Guangzhou 511430, China; caesar@chimelong.com; 4 Southern China Wildlife Species Conservation Center, Zhuhai 519031, China

**Keywords:** giraffe, daily behaviors, well-being, keypoint, spatial-adaptive two-stream network

## Abstract

The daily behavioral patterns of giraffes reflect their health status and well-being. Behaviors such as licking, walking, standing, and eating are not only essential components of giraffes’ routine activities but also serve as potential indicators of their mental and physiological conditions. This is particularly relevant in captive environments such as zoos, where certain repetitive behaviors may signal underlying well-being concerns. Therefore, developing an efficient and accurate automated behavior detection system is of great importance for scientific management and welfare improvement. This study proposes a multi-behavior automatic detection method for giraffes based on YOLO11-Pose and the spatial-adaptive two-stream network (SATSN). Firstly, YOLO11-Pose is employed to detect giraffes and estimate the keypoints of their mouths. Observation-Centric SORT (OC-SORT) is then used to track individual giraffes across frames, ensuring temporal identity consistency based on the keypoint positions estimated by YOLO11-Pose. In the SATSN, we propose a region-of-interest extraction strategy for licking behavior to extract local motion features and perform daily behavior classification. In this network, the original 3D ResNet backbone in the slow pathway is replaced with a video transformer encoder to enhance global spatiotemporal modeling, while a Temporal Attention (TA) module is embedded in the fast pathway to improve the representation of fast motion features. To validate the effectiveness of the proposed method, a giraffe behavior dataset consisting of 420 video clips (10 s per clip) was constructed, with 336 clips used for training and 84 for validation. Experimental results show that for the detection tasks of licking, walking, standing, and eating behaviors, the proposed method achieves a mean average precision (mAP) of 93.99%. This demonstrates the strong detection performance and generalization capability of the approach, providing robust support for automated multi-behavior detection and well-being assessment of giraffes. It also lays a technical foundation for building intelligent behavioral monitoring systems in zoos.

## Simple Summary

We propose a deep learning-based method for detecting daily behaviors in giraffes, aiming to improve detection accuracy and support zoo staff in more effectively monitoring giraffe behavior. This approach promotes a more scientific and proactive strategy for monitoring giraffe health and enhancing their physical and psychological well-being. A spatial-adaptive two-stream network is proposed to reduce false positives and missed detections. Experimental results demonstrate that the method achieves high accuracy and stability in detecting giraffe daily behaviors, offering an effective technological solution for long-term, non-contact, and intelligent behavior monitoring with promising application potential.

## 1. Introduction

Animal well-being is an important criterion for evaluating zoological institutions. Physical well-being has long been considered a core component of the well-being of captive animals, while psychological well-being has increasingly been recognized as an essential aspect of overall animal well-being over time [1]. Giraffes are common large herbivores in zoological settings and possess significant economic and scientific value. Their daily behavioral patterns serve as important indicators for assessing individual physiological health and psychological state [2]. For instance, changes in standing duration, eating frequency, and the frequency of licking non-nutritive objects may reflect potential behavioral abnormalities or psychological stress. Under captive conditions, giraffes are frequently observed engaging in repetitive licking of non-food items such as trees, enclosures, or eating apparatuses.

Oral stereotypic behavior has been extensively studied in domesticated ungulates [3,4]. Stereotypic behavior is defined as a repetitive, invariant behavioral pattern with no apparent goal or function [5], typically developed as a coping mechanism in response to restrictive environments [6,7]. This behavior is widely regarded as an indicator of poor animal well-being [8,9]. Stereotypic behaviors can lead to reducing performance in sport [10,11] and various health issues, including digestive disorders, weakened immune function, and in severe cases, reduced reproductive capability [12]. Observations at Chimelong Safari Park indicate that the giraffe population generally exhibits natural behavioral expressions, with only a small number of individuals displaying stereotypic behaviors. Therefore, the timely and automated detection of giraffe licking behavior can assist zoo management in studying oral-related behaviors and contribute to the prevention of potential stereotypic behavior development.

Traditional behavior detection methods often rely on manual observation, which is labor-intensive and time-consuming. Additionally, observer variability may introduce biases in the results [13]. In recent years, numerous researchers have adopted sensor-based approaches for animal behavior detection. For example, wireless sensor nodes attached to pigs have been utilized to collect data for behavior monitoring [14]. Similarly, accelerometers mounted on cattle have been employed to capture movement data and identify behavioral patterns [15]. In lamb, halter-mounted accelerometer sensors have been used for data collection and behavior detection [16]. However, these methods may interfere with animals’ natural behaviors, potentially causing stress or excessive reactions, which can compromise data accuracy and maybe raise animal well-being concerns.

With the rapid advancement of computer vision in recent years, an increasing number of researchers have applied computer vision techniques for animal behavior detection. Hakansson et al. [17] employed Convolutional Neural Networks (CNN) and Long Short-Term Memory (LSTM) to detect tail-biting behavior in pig herds. Kaifeng et al. [18] developed a two-stream convolutional network based on an Inflated 3D Convolutional Neural Network and Temporal Segment Network to detect behaviors such as eating, lying down, walking, scratching, and mounting in groups of pigs. Li et al. [19] implemented sow nursing behavior recognition based on the SlowFast and hidden Markov models. Tu et al. conducted research on group-housed pig tracking and behavior analysis based on YOLOv8 and OC-SORT [20].

Although animal behavior detection technologies have advanced in recent years, existing studies have primarily focused on behaviors with salient features and large motion amplitudes. In contrast, the detection of daily behaviors in giraffes remains a significant challenge. Taking licking behavior as an example, its subtle motion and minimal body displacement make it difficult to detect accurately in image sequences. The proposed method effectively addresses the challenges of traditional manual observation methods, such as high time consumption and subjective data interpretation, while also avoiding potential interference from wearable sensor devices that could affect animal behavior. The main contributions of this study are as follows:

I. We propose an automated multi-behavior detection method for giraffes, integrating object detection, keypoint estimation, multi-object tracking, and behavior detection modules. In addition, a mouth-region-based strategy is designed specifically for detecting licking behavior. The proposed method enables efficient recognition of various daily behaviors, including licking, eating, standing, and walking.
II. In the behavior detection module, the SlowFast network is structurally enhanced to improve spatiotemporal modeling capabilities. Specifically, a Video Transformer (ViT) encoder is integrated into the slow pathway to strengthen the modeling of long-range spatiotemporal dependencies, while a Temporal Attention (TA) module is embedded in the fast pathway to improve responsiveness to motion changes. These architectural modifications significantly enhance the model’s ability to improve classification accuracy.
III. Experimental results demonstrate the effectiveness of the proposed method in detecting giraffe daily behaviors within zoo environments. This study introduces a novel approach for monitoring such behavior, offering practical value for the timely surveillance and scientific management of giraffes’ physical and psychological well-being in zoological settings.

## 2. Methods

### 2.1. Materials

#### 2.1.1. Animals and Video Acquisition

The video data were collected from October 2024 to February 2025 at Chimelong Safari Park in Guangzhou, using RGB video cameras from four different viewpoints. Recordings were conducted under both sunny and overcast weather conditions. After screening for clarity—eliminating footage that was either too blurry or lacked sufficient visibility—420 videos were selected for analysis. The giraffes featured in the videos were located in the zoo’s public exhibition area, where they could move freely and interact with other animals such as equus quagga and taurotragus oryx within the same enclosure. In total, 75 giraffes (29 northern giraffes and 46 reticulated giraffes) were included in the sample, comprising both males and females, with ages ranging from 1 to 22 years. The videos were recorded using fixed cameras at a frame rate of 30 frames per second with a resolution of 3840 × 2160 pixels and stored in MP4 format.

#### 2.1.2. Data Preprocessing

**Figure 1:**
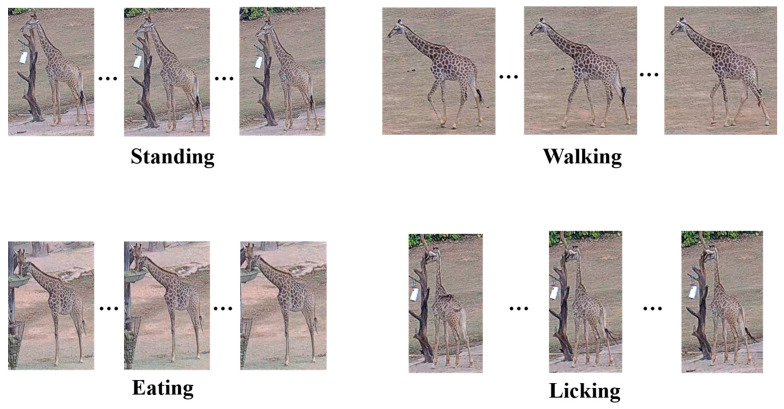
Schematic of giraffe daily behaviors.

This study constructed two types of datasets. The first is a YOLO [21] format dataset for giraffe detection, used to train and evaluate the detection and localization of giraffes. The second is an AVA-format dataset [22] for giraffe behavior detection, designed to train and test the detection of giraffes engaging in daily behavior. From approximately 30 h of video footage, 2200 images were extracted for the giraffe detection dataset and annotated using the Software Labelme (version 5.5.0, https://github.com/wkentaro/labelme.git accessed on 10 October 2024) to label both the bounding boxes for giraffes and the keypoints corresponding to their mouths. The giraffe detection dataset was divided into training, validation, and test sets in a ratio of 8:1:1, resulting in 1760 training images, 220 validation images, and 220 test images. The giraffe behavior detection dataset consisted of 420 video clips, each with a duration of 10 s. These were split into 336 clips for training and 84 clips for testing, following an 8:2 ratio. Behavioral annotations were performed using the VIA (VGG Image Annotator) tool and subsequently converted into the AVA format. Data of training set was augmented by rotation, color jittering, and flipping to improve the data generalization. The giraffe behaviors [2,23,24] are shown in Figure 1 and the behaviors description in Table 1.

**Figure 2:**
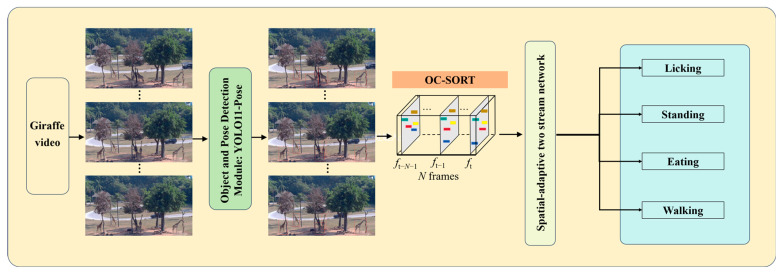
The workflow of the proposed method. YOLOv11-Pose is used to detect giraffes and locate their mouth keypoints, OC-SORT performs tracking across frames, and the Spatial-adaptive two stream network classifies giraffe daily behaviors.

We propose an improved giraffe daily behaviors detection framework, which integrates object detection, keypoint estimation, multi-object tracking, and behavior detection. The overall workflow is illustrated in Figure 2.

First, YOLOv11-Pose is used to detect giraffes and locate their mouth keypoints. Then, OC-SORT is applied to perform multi-object tracking, assigning a unique ID to each giraffe to ensure consistent identity across video frames. Building upon this, we introduce Spatial-adaptive two stream network that includes a region-of-interest extraction strategy for licking behavior based on keypoint localization. If a giraffe is identified as performing a licking behavior, the cropped mouth region is used as the input bounding box in fast path; otherwise, the full-body detection box is used in slow path. In this framework, the slow pathway replaces the original three-dimensional Residual Network (3D ResNet) [25] with a ViT encoder, enhancing the modeling of global spatial–temporal dependencies. Meanwhile, the fast pathway incorporates a Temporal Attention (TA) module to improve sensitivity to rapid motion features. This hybrid architecture combines the global modeling capability of Transformers with the multi-scale dynamic perception of the SlowFast [26] framework, significantly enhancing the detection performance for giraffe behaviors such as licking, standing, walking, and eating.

### 2.2. YOLO11-Pose Network

**Figure 3:**
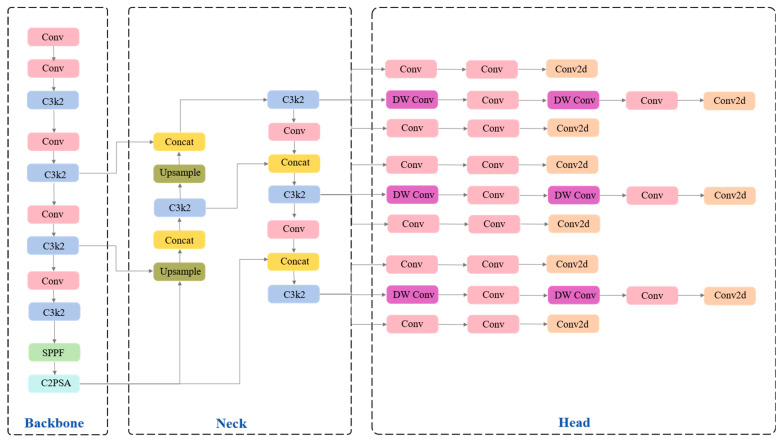
The structure of YOLO11-Pose.

YOLO11-Pose is a fast and efficient object detection and keypoint estimation model extended from YOLO11 [27], well-suited for real-time computer vision tasks. In this study, YOLO11-Pose is adopted as the detection network. It can accurately locate giraffes and their mouth keypoints, based on which we propose a region-of-interest extraction strategy for licking behavior driven by keypoint localization. The model not only inherits the advantages of previous YOLO versions but also introduces innovations in structural design and functional modules. Specifically, YOLO11-Pose enhances the feature extraction capability, enabling the model to capture fine-grained local and structural details in images. While introducing a dedicated keypoint prediction branch, the overall architecture remains compact, and both detection and pose estimation accuracy are significantly improved. The overall architecture of YOLO11-Pose is illustrated in Figure 3.

A Backbone, a Neck, and a Head are incorporated into the model’s architecture. The Backbone is composed of Conv, C3k2, SPPF, and C2PSA modules, which together facilitate multi-scale feature extraction. The C3k2 module improves processing efficiency through two lightweight convolutions; the SPPF module enhances the ability to model multi-scale objects; and the C2PSA module strengthens the model’s focus on key regions through spatial attention mechanisms. The Neck integrates features from different scales using Upsampling, Concat, and additional C3k2 modules, effectively enhancing the model’s ability to recognize small objects and keypoints, thereby improving robustness in both detection and pose estimation tasks. The Head, composed of DWConv, Conv, and Conv2d layers, is responsible for outputting bounding boxes, class labels, and the mouth keypoints of giraffes.

### 2.3. Spatial-Adaptive Two Stream Network

**Figure 4:**
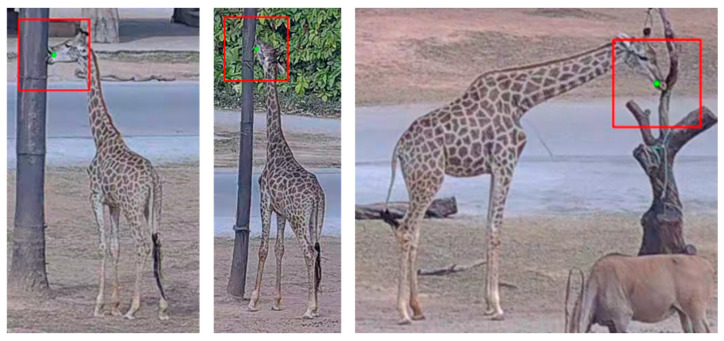
The illustrated of extract regions. The 128 × 128 pixels cropped regions are generated based on the keypoint positions, which enable the model to focus on behavior-relevant features during the detection of licking behavior.

Giraffe licking and eating behaviors both involve movements of the mouth, but they differ significantly in terms of overall body posture. According to our observations, the giraffes at Chimelong Safari Park usually exhibit obvious bodily movements when eating, showing strong global postural characteristics; whereas body posture during licking behavior is less fixed, with key discriminative cues primarily concentrated in the mouth region. To further enhance the detection accuracy of giraffe licking behavior and systematically explore the impact of input granularity on model performance, we propose a Spatial-adaptive two stream network based on the SlowFast (pretrained on the Kinetics-400 dataset) that incorporating a localized region feature extraction strategy. Specifically, we construct local image patches of size 128 × 128 pixels centered on the mouth keypoints detected by YOLO11-Pose, a choice substantiated by experimental evidence demonstrating its superior effectiveness. These cropped regions, illustrated in Figure 4, enable the model to focus on behavior-relevant features while reducing background noise.

This approach aims to enhance the model’s focus on and representation of this critical region. Within the SATSN architecture, we design three different input combinations to evaluate the effectiveness of localized inputs. In the first configuration, the fast pathway receives the cropped mouth region, while the slow pathway processes the full-body image of the giraffe. This setting leverages the fast pathway’s high temporal resolution to capture fine-scale oral motion patterns, while the slow pathway provides complementary modeling of overall body posture and global context. In the second configuration, the slow pathway is fed with the cropped mouth region, whereas the fast pathway continues to receive the full-body image. This design aims to enhance the slow pathway’s capacity for modeling localized semantic cues within the mouth region, while the fast pathway captures rapid full-body dynamics. All cropped regions are automatically generated based on the keypoint positions, with a fixed size of 128 × 128 pixels to ensure adequate coverage of behavior-relevant features. It is important to note that this differentiated input strategy is applied exclusively to samples labeled as licking behavior, whereas samples corresponding to other behaviors continue to use the full-body bounding box images as input for both the slow and fast pathways.

**Figure 5:**
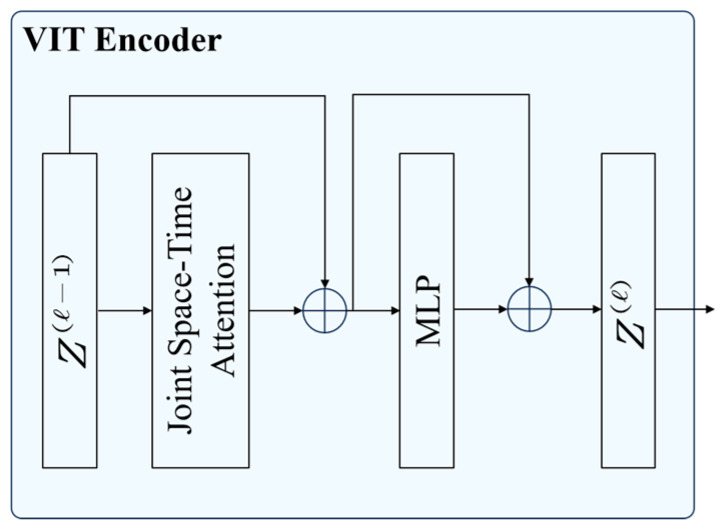
The structure of ViT encoder.

The proposed framework adopts a dual-pathway architecture, consisting of a slow pathway and a fast pathway, both originally employing 3D ResNet as their backbone network. While the 3D ResNet in the slow pathway provides strong modeling capacity for spatial–temporal features, the daily behaviors of giraffes are typically characterized by slow movements and small motion amplitudes, rendering such a heavy backbone partially redundant and leading to increased inference latency. To address this, we replace the 3D ResNet in the slow pathway with a ViT encoder, aiming to enhance feature representation efficiency while reducing computational overhead. The architecture of the ViT encoder is illustrated in Figure 5.

**Figure 6:**
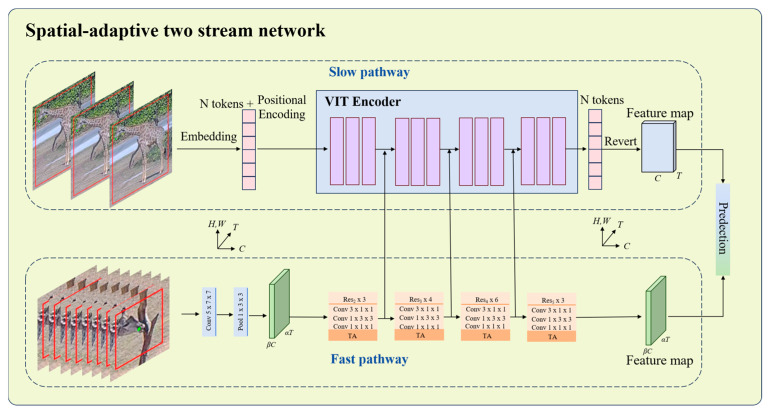
The structure of SATSN. The fast pathway receives the cropped mouth region, while the slow pathway processes the full-body image of the giraffe.

The ViT encoder used in this study is adopted from Vivit [28] with Joint Space–Time Attention [29], Z represents the embedding vectors derived from tubelet tokens. Compared with 3D ResNet, ViT encoder provides better parameter efficiency, helping to reduce redundant computation and improve inference speed while maintaining high accuracy. In addition, the ViT encoder leverages self-attention mechanisms to model global spatiotemporal dependencies, enabling comprehensive extraction of dynamic features across different behaviors. This makes it suitable for fine-grained modeling of giraffe behaviors that involve subtle and slow movements. The architecture of the SATSN is illustrated in Figure 6.

In this architecture, *H* and *W* denote the spatial resolution height and width, respectively, while *T* and *C* denote the frame count and channel count; *α* denotes the relative frame rate of the Fast pathway to the Slow pathway (*α* > 1), while *β* describes the proportion of channels allocated to the Fast pathway compared to the Slow one (*β* < 1); and *τ* represents the temporal stride.

In the Slow pathway, the input sequence consists of T frames sampled with a temporal stride of *τ.* In contrast, the Fast pathway samples *α*T frames, which is α times denser than the Slow pathway, and uses fewer channels (*β*C) along with smaller convolutional kernels, thereby reducing its capacity for spatial representation. Feature fusion is achieved through several lateral connections from the Fast to the Slow pathway. Specifically, the output features from each Res block in the Fast pathway are reshaped to match the feature dimensions in the Slow pathway, enabling lateral integration across stages.

### 2.4. TA (Temporal Attention) Module

**Figure 7:**
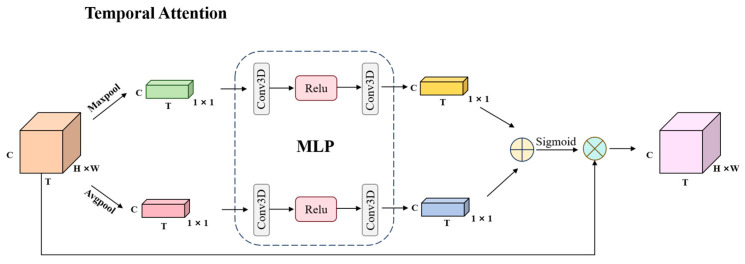
The structure of Temporal Attention.

The input $X\;\in \;{\mathbb{R}}^{T\times C\times H\times W}$ is first compressed using average pooling and max pooling operations along the temporal dimension. These average pooling and max pooling outputs are then processed by a shared layer MLP, which consists of two 3D convolutional layers with convolution kernel of size 1, and a relu layer. The $W_{m}\in {\mathbb{R}}^{T\times \frac{T}{r}}$ and $W_{n}\in {\mathbb{R}}^{\frac{T}{r}\times T}$ denote the weights of the first and second convolutional layers, respectively. The parameter *r* controls the reduction in the temporal dimension to limit computational cost. The output of the shared layer MLP is formulated as Equation (1),

(1) $$F_{T}\left(X\right)=(W_{m}\left(ReLU\left(W_{n}\left(AvgPool\left(X\right)\right)\right)\right)+W_{m}\left(ReLU\left(W_{n}\left(MaxPool\left(X\right)\right)\right)\right)$$

where $F_{T}\left(X\right)$ denotes the process carried out by the temporal attention. After passing through the MLP, the average pooling and max pooling outputs are transformed into a temporal weight value $M\in R^{T}$. The detailed process is illustrated in Figure 7.

### 2.5. OC-SORT

The movements of giraffes are relatively slow and exhibit complex trajectory variations, which are often accompanied by prolonged occlusions and overlaps. In this study, OC-SORT [30] is employed as the tracking method for giraffe. This approach introduces three key improvements: Observation-Centric Re-Update (ORU), Observation-Centric Momentum (OCM), and Observation-Centric Recovery (OCR), which significantly enhance tracking accuracy and robustness under conditions of occlusion, slow motion, and complex trajectory changes.

ORU: When an object is re-detected after an occlusion (i.e., the trajectory is re-activated), the system constructs a virtual trajectory ${\tilde{Z}}_{t}$ based on the last observation before occlusion $Z_{t_{1}}$ and the newly observed detection $Z_{t_{2}}$. This virtual trajectory effectively compensates for the accumulated prediction error during the occluded period. The expression of the virtual trajectory is as follows:

(2) $${\tilde{Z}}_{t}=Traj_{virtual}\left(z_{t_{1}},z_{t_{2}},t\right),\;t_{1}<t<t_{2}$$

Subsequently, a Kalman Filter update is performed along the virtual trajectory, which effectively suppresses the accumulated drift in prediction during occlusion, thereby enhancing the stability and accuracy of trajectory recovery.

(3) $$\mathrm{re}\text{-}\mathrm{update}\{\begin{array}{c} K_{t}=P_{t|t-1}H_{t}^{⊤}{\left(H_{t}P_{t|t-1}H_{t}^{⊤}+R_{t}\right)}^{-1}\\ {\hat{X}}_{t|t}={\hat{X}}_{t|t-1}+K_{t}\left({\tilde{z}}_{t}-H_{t}{\hat{X}}_{t|t-1}\right)\\ P_{t|t}=\left(I-K_{t}H_{t}\right)P_{t|t-1} \end{array}$$

OCM: This enhancement computes the motion direction of a trajectory based on direct observations rather than estimated states and incorporates directional consistency into the matching cost function as a key criterion for assessing the validity of associations. By evaluating the angular deviation between the historical trajectory direction and the current observed direction, OCM is able to more accurately model the motion tendency of the object, thereby significantly reducing the incidence of identity switches.

(4) $$C\left(\hat{X},Z\right)=C_{\mathrm{IoU}}\left(\hat{X},Z\right)+\lambda C_{v}\left(Z,Z\right)$$

OCR: Following the main association stage, an additional matching step based on Generalized Intersection over Union is introduced to attempt re-association between unmatched trajectories from the previous frame and current detections. This improvement enhances the system’s ability to recover object experiencing short-term occlusion, stationary behavior, or low detection confidence, thereby increasing overall matching recall and tracking continuity.

### 2.6. Setup

The experimental environment in this study consisted of a server equipped with a single NVIDIA GeForce RTX 2080 Ti GPU with 11 GB of memory and an Intel Core i7-7700 CPU processor running at 3.60 GHz. The experiments were conducted using the PyTorch deep learning framework (version 2.1.2) on the Ubuntu 20.04 operating system, with Python 3.8 as the programming language. The training hyperparameters for the experiments are shown in Table 2.

## 3. Results

### 3.1. Evaluation Metrics

In this study, evaluation metrics commonly used in behavior detection tasks—precision, recall, and *F*1*-measure* [31]—are employed to assess the performance of the proposed method for detecting giraffes daily behaviors. Precision measures the proportion of true positive instances among all instances predicted as daily behaviors, indicating the accuracy of the model’s positive predictions. Recall reflects the proportion of actual daily behaviors that are correctly identified, representing the model’s ability to detect relevant behaviors. The *F*1*-measure*, as the harmonic mean of precision and recall, provides a balanced evaluation of both accuracy and completeness, and serves as a comprehensive metric for overall detection performance. Average Precision (AP) is computed as the area under the Precision–Recall curve, and serves as a key metric for evaluating the accuracy of behavior detection in each category. The mean Average Precision (mAP) is calculated by averaging the AP values across the four defined giraffe behavior categories: standing, licking, eating, and walking. In addition, we evaluated model size as an indicator of computational cost, measured by the total storage required for the trained model parameters (in megabytes, MB).

(5) $$Precision=\frac{TP}{TP+FP}\times 100\%$$

(6) $$Recall=\frac{TP}{TP+FN}\times 100\%$$

(7) $$F1\text{-}measure=\frac{2Recall\cdot Precision}{Recall+Precision}\times 100\%$$

(8) $$AP=\int_{0}^{1}P\left(R\right)dR$$

(9) $$mAP=\frac{1}{k}\sum_{i=1}^{k}AP$$

where *TP* (True Positives), *FP* (False Positives), and *FN* (False Negatives) denote the number of behavior of giraffes that are correctly detected, incorrectly detected, and missed, respectively.

### 3.2. Experimental Results

To evaluate the impact of the proposed keypoint-guided localized region cropping and pathway input strategy on licking behavior detection, three input configurations were tested on the constructed dataset: feeding full-body images into both pathways (A), feeding the localized mouth region into the fast pathway and the full-body image into the slow pathway (B), and feeding the localized mouth region into the slow pathway and the full-body image into the fast pathway (C). The experimental results are presented in Table 3.

The results show that all three localized pathway input strategies improved the detection performance of licking behavior to varying degrees. Among them, configuration B achieved the best performance across all evaluation metrics, with the AP increasing to 91.69%, Precision and Recall reaching 92.33% and 91.29%, respectively, and the F1-measure reaching 91.81%, representing a significant improvement over the full-body input baseline A, demonstrating the method’s effectiveness in the task of giraffe licking behavior detection.

**Figure 8:**
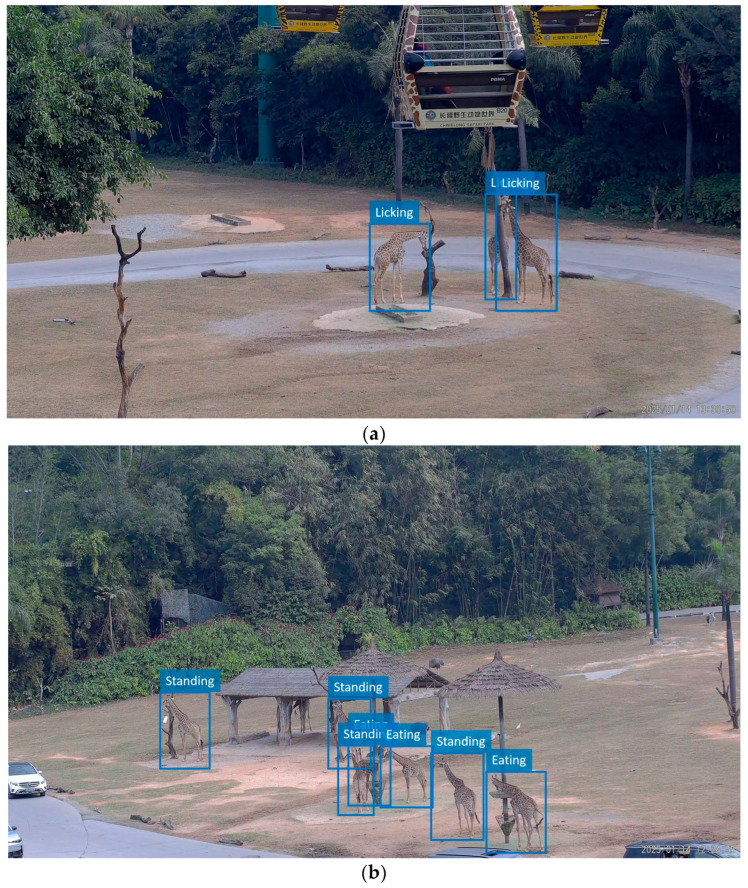

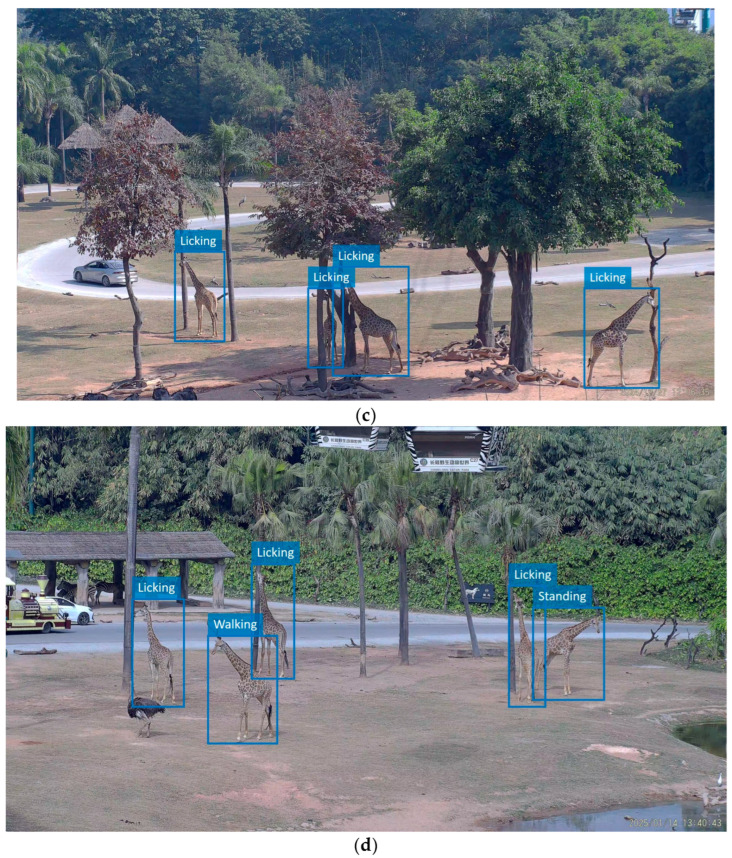
Detection results of the giraffe daily behaviors, (**a**–**d**) correspond to different camera viewpoints.

Building upon the detailed analysis of licking behavior, we further evaluated the proposed method across all annotated giraffe behaviors to assess its overall effectiveness. The results show that the AP for the other three behavior categories—standing, walking, and eating—reached 92.30%, 95.90%, and 96.08%, respectively, with an overall mAP of 93.99%. These results indicate that the proposed method is effective in detecting various giraffe behaviors. Figure 8 illustrates the visualization results of detecting daily behaviors.

**Figure 9:**
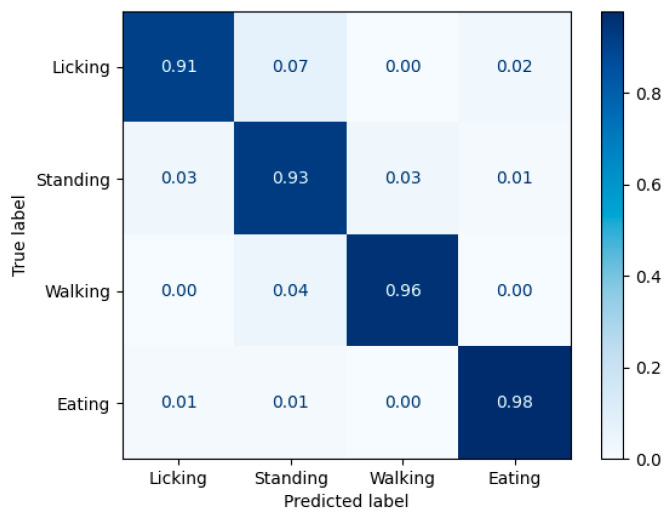
The confusion matrix of the behavior detection results.

Notably, the AP for licking is significantly lower than that of the other behaviors. As shown in the confusion matrix (Figure 9), misclassifications occur across several behavior categories, with the most frequent confusion between licking and standing, which may be attributed to occlusions caused by elements such as trees.

In particular, to further validate the method’s discriminative capability under challenging conditions, we selected a subset of test video clips in which the distance between the giraffe’s mouth and the tree surface was zero. These clips contained both licking and non-licking intervals. Under this setting, the proposed method achieved a precision of 92.31% in distinguishing licking behavior from non-licking behavior, demonstrating its strong ability in fine-grained behavior detection.

### 3.3. Ablation Experiments and Comparison Different Behavioral Detection Methods

To systematically evaluate the effectiveness of the proposed architectural modifications—namely replacing the 3D ResNet in the slow pathway with a ViT encoder and embedding the TA module into the fast pathway—this study conducted a series of ablation experiments based on a self-constructed giraffe behavior dataset. The original SlowFast model was used as the baseline, while the ViT encoder and TA module were introduced individually and then jointly, in order to investigate their respective and combined impacts on the overall model performance. The experimental results are presented in Table 4.

The second row shows the results obtained by incorporating the TA module into the fast pathway. Compared with the baseline (SlowFast), the mAP of giraffe daily behaviors increased by 0.55%. These improvements suggest that the TA module effectively enhances the model’s ability to focus on behavioral features in temporal dimensions.

The third row displays the results of replacing the 3D ResNet in the slow pathway with a ViT encoder. Compared with the baseline, the mAP of giraffe daily behaviors increased by 1.42%. These findings indicate that the ViT encoder enhances the model’s ability to capture long-range spatial–temporal dependencies, thereby improving its capacity to model complex motion patterns across all four giraffe behaviors.

The fourth row reports the results of the model in which both the TA and ViT encoder were applied. Compared with the baseline, the mAP of giraffe daily behaviors increased by 4.41%. And our method achieves the highest speed of 5.7 fps. This result demonstrates the effectiveness of the proposed method.

This study conducted comparative experiments on a self-constructed giraffe behavior dataset, selecting SlowOnly, ACRN [32], and VideoMAE [33] as benchmark models. The results are shown in Table 5. Table 5 shows that the proposed method achieves superior performance compared to the other methods.

### 3.4. Giraffe Daily Behaviors Statistics

**Figure 10:**
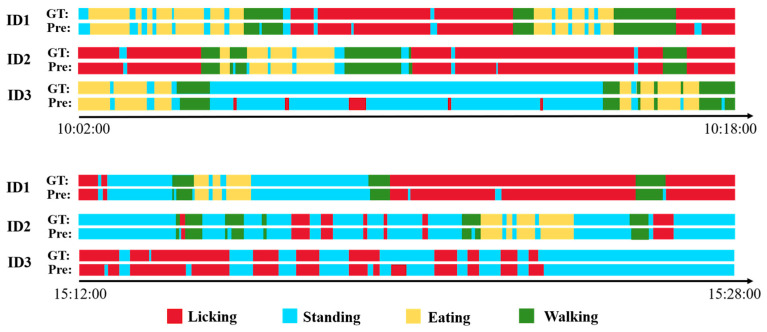
The statistics of daily giraffe behaviors in sampled test cases. GT refers to the ground truth, and Pre indicates the predicted results by our method.

To evaluate the effectiveness of the proposed method in behavior detection, three giraffes were randomly selected, and their behavioral patterns were analyzed during two specific time intervals: 10:02–10:18 in the morning and 15:12–15:28 in the afternoon. As illustrated in Figure 10, the proposed approach demonstrates a high level of accuracy in detecting daily giraffe behaviors. However, some misclassifications were observed. For instance, certain instances of licking behavior were misidentified as standing due to occlusions caused by trees. Moreover, the experimental data show that standing and licking are the two behaviors with the longest durations, and that eating activity was significantly more frequent in the morning compared to the afternoon.

**Figure 11:**
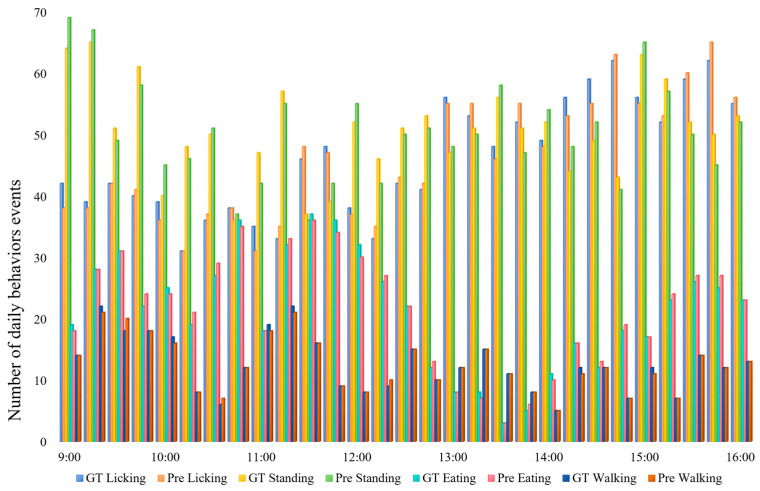
The statistics of daily giraffe behaviors recorded between 9:00 and 16:00.

To gain a broader understanding of behavioral patterns, we further extended the observation window to the 9:00–16:00 period and quantified the number of behavior events for each category. As illustrated in Figure 11, this analysis provides a comprehensive overview of the occurrence frequency of different behaviors, offering deeper insights into the daily activity patterns of giraffes. Notably, the frequency of licking behavior is higher in the afternoon compared to the morning, while eating behavior is observed more frequently between 10:00 and 12:00.

## 4. Discussion

To the best of our knowledge, this study is the first to detect giraffe daily behaviors based on computer vision. Our method not only overcomes the limitations of traditional manual observation in terms of efficiency, but also provides a technical foundation for long-term monitoring of behavioral patterns and individual health assessment. Developing a logically structured and practically feasible solution for automatic behavior identification is practically achievable.

### 4.1. Advantages of SATSN

The proposed framework offers a comprehensive solution for giraffe daily behavior detection by integrating object detection, keypoint estimation, multi-object tracking, and a Spatial-adaptive two-stream network with region-of-interest extraction. In particular, we propose a local feature extraction strategy that adaptively crops keypoint-centered mouth regions to effectively capture subtle and fine-grained motion cues. This design effectively addresses the unique challenge that giraffe behaviors often involve subtle mouth movements and minimal overall body motion, which are difficult to detect using conventional vision-based techniques. Furthermore, the slow pathway replaces the original 3D ResNet with a ViT encoder, enhancing global spatial–temporal modeling, while the fast pathway incorporates a TA module to improve sensitivity to rapid motion changes. The method achieves promising results in giraffe daily behavior detection, with APs of 91.69%, 92.30%, 95.90%, and 96.08% for licking, standing, walking, and eating behaviors, respectively, and an overall mAP of 93.99%, underscoring its effectiveness in giraffe daily behavior detection tasks. It can be concluded from Table 4 that the higher inference speed of SATSN compared with the base model is attributed to the incorporation of the localized region feature extraction strategy, which drastically reduces redundant computations by focusing on the mouth-centered regions of interest. Specifically, lower-resolution video frames replace the fast pathway inputs, so that a larger portion of the input data is processed at reduced resolution, further decreasing the computational load.

### 4.2. Performance Analysis

As illustrated in Table 5, the proposed SATSN method has a model size of approximately 332 MB. In comparison, SlowOnly is smaller at about 258 MB, as it relies solely on the slow pathway and simplifies the original SlowFast architecture. However, this simplification leads to a clear trade-off in performance, as SlowOnly struggles to capture fine-grained behavioral details. ACRN, with a substantially larger model size of around 737 MB, leverages 3D convolutions to extract spatiotemporal features and focuses on modeling interactions between the central actor and surrounding objects. VideoMAE, based on the Video Transformer architecture, has the largest model size among all compared methods at approximately 1,064 MB, employing a masked autoencoding strategy to learn latent spatiotemporal and semantic representations. Although SATSN is moderately larger than SlowOnly, it delivers a substantially higher mean average precision. Moreover, despite being smaller than ACRN and significantly smaller than VideoMAE, SATSN still achieves superior accuracy, demonstrating an optimal trade-off between model size and detection performance. Note that four methods detect giraffe walking behavior at high AP as walking behavior is distinct among the four behaviors.

The proposed SATSN replaces the 3D ResNet in the slow pathway with a ViT encoder and embeds the TA module into the fast pathway, significantly enhancing the model’s capacity to capture temporal dependencies in behavioral sequences. More importantly, SATSN is designed as a spatial-adaptive two-stream network with region-of-interest extraction, enabling the integration of both global contextual representations and local fine-grained features, making it particularly well-suited for detecting giraffe daily behaviors.

Among the four types of giraffe daily behaviors, licking behavior is the most challenging to detect. This difficulty arises because when a giraffe’s head is positioned close to a tree, it may simply be standing rather than performing a licking action, which often leads to misclassification. Our proposed method effectively addresses this issue by accurately distinguishing between these subtle behavioral states, thereby improving the reliability of behavior detection.

### 4.3. Limitations

**Figure 12:**
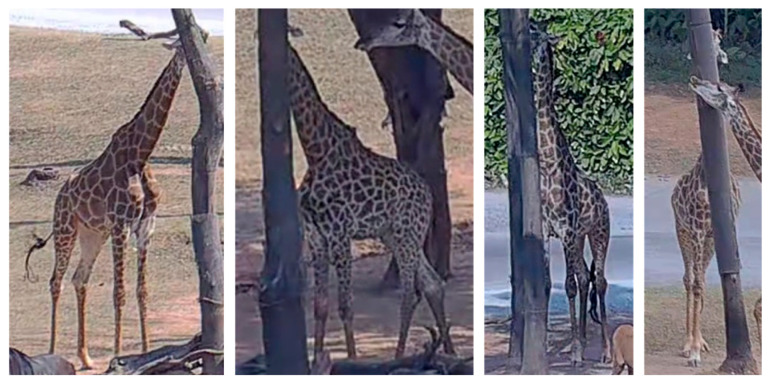
Failure cases where giraffe mouths are occluded.

Although the proposed method demonstrates promising performance in detecting daily behaviors in giraffes, its effectiveness may be constrained under zoo environments, where such behavior is frequently subject to varying degrees of occlusion. Giraffes may occlude each other during interactions, and environmental elements such as tree branches, feeding containers, and fences can further obstruct the camera’s field of view. As illustrated in Figure 12, the giraffe’s mouth—critical for identifying the giraffe behavior—may not be fully captured in the video frames. However, occlusions and environmental obstructions are general challenges in the field of computer vision, which are not unique to SATSN.

These occlusions lead to incomplete feature representations being fed into the model, which limits its capacity for temporal modeling and fine-grained motion analysis, ultimately increasing the risk of missed detections or false positives.

In addition to the challenges caused by occlusions, another limitation of the proposed methodology is that it is restricted to group-level behavioral detection and cannot provide assessments at the individual level. In future work, individual marking could be considered as a potential approach to enable individual-level behavioral assessment.

## 5. Conclusions

The SATSN proposed in this study successfully achieves efficient detection of daily behaviors in giraffes within zoo environments, providing a novel technical solution for the detection of giraffe daily behaviors. This method can assist animal experts in evaluating the health status of giraffes based on their behaviors, thereby enhancing the overall supervision capabilities of zoological institutions.

In this study, YOLOv11-Pose is employed to detect giraffes and localize keypoints around the mouth region. OC-SORT is then used for multi-object tracking of individual giraffes. Then we propose a region-of-interest extraction strategy for licking behavior to extract local motion features and perform daily behavior classification.

Experimental results demonstrate that the SATSN achieves an overall mAP of 93.99% in detecting giraffe daily behaviors. These findings indicate that the proposed method enables accurate identification of daily behaviors. Comparative experiments against existing behavior detection methods reveal that the SATSN offers notable advantages, thereby validating the effectiveness and reliability of the proposed approach in practical applications. Consequently, integrating this approach with manual observation not only provides a valuable reference for the detection of giraffe daily behaviors, but also strengthens the management of giraffes in zoological institutions, ultimately contributing to animal well-being.

## Figures and Tables

**Table 1:** The description of giraffe daily behaviors.

Behavior	Behavior Description
Licking	Movement of the tongue across trees, troughs, or other non-food objects.
Standing	Maintains both forelimbs and both hindlimbs to support the body and does not engage in any of the other behaviors listed.
Walking	Significant change in body position such as moving in a normal position.
Eating	Ingestion of food in troughs or nibbling low on ground forage.

**Table 2:** The training hyperparameters.

Hyperparameters	Batch Size	Epochs	Learning Rate	Momentum
Value	4	200	0.02	0.9

**Table 3:** The result of different input paths on licking behavior (reported as mean ± standard deviation over five experiments).

Different Input Scheme	AP	*Precision*	*Recall*	*F1-Measure*
A	88.38% ± 0.10	88.90% ± 0.07	88.38% ± 0.05	88.63% ± 0.05
B	91.69% ± 0.27	92.33% ± 0.14	91.29% ± 0.23	91.81% ± 0.16
C	87.70% ± 0.09	87.24% ± 0.10	89.50% ± 0.11	88.36% ± 0.05

**Table 4:** The results of ablation experiments.

Model	Description	*mAP*	Model Size (MB)	Speed (FPS)
SlowFast	Original SlowFast architecture	89.58%	273	4.2
SlowFast + TA	Integrated TA module into the fast pathway	90.13%	289	2.3
SlowFast + VIT Encoder	Replaced the slow pathway’s 3D ResNet with ViT encoder	91.00%	323	4.9
SATSN (Ours)	ViT encoder in slow pathway + TA module in fast pathway + localized region feature extraction strategy	93.99%	332	5.7

**Table 5:** The comparative results of different behavioral detection methods (reported as mean ± standard deviation over five experiments).

Model	*mAP*	*Licking* (*AP*)	*Standing* (*AP*)	*Walking* (*AP*)	*Eating* (*AP*)	Model Size (MB)
SATSN (Ours)	93.99% ± 0.01	91.69% ± 0.27	92.30% ± 0.07	95.90% ± 0.09	96.08% ± 0.12	332
SlowOnly	89.02% ± 0.13	85.82% ± 0.19	87.38% ± 0.18	93.85% ± 0.09	89.25% ± 0.23	258
ACRN	85.87% ± 0.12	73.39% ± 0.16	86.70% ± 0.16	96.24% ± 0.15	87.35% ± 0.19	737
VideoMAE	89.42% ± 0.24	84.77% ± 0.14	87.64% ± 0.15	96.01% ± 0.11	90.06% ± 0.11	1064

## Data Availability

The data presented in this study are available on request from the corresponding authors.

## References

[1] Bashaw M.J., Tarou L.R., Maki T.S., Maple T.L. (2001). A Survey Assessment of Variables Related to Stereotypy in Captive Giraffe and Okapi. Appl. Anim. Behav. Sci..

[2] Wark J.D., Cronin K.A. (2024). The Behavior Patterns of Giraffes (*Giraffa camelopardalis*) Housed across 18 US Zoos. PeerJ.

[3] Terlouw E.M.C., Lawrence A.B., Illius A.W. (1991). Influences of Feeding Level and Physical Restriction on Development of Stereotypies in Sows. Anim. Behav..

[4] Redbo I. (1990). Changes in Duration and Frequency of Stereotypies and Their Adjoining Behaviours in Heifers, before, during and after the Grazing Period. Appl. Anim. Behav. Sci..

[5] Mason G.J. (1991). Stereotypies: A Critical Review. Anim. Behav..

[6] Bandeli M., Mellor E.L., Kroshko J., Maherali H., Mason G.J. (2023). The Welfare Problems of Wide-Ranging Carnivora Reflect Naturally Itinerant Lifestyles. R. Soc. Open Sci..

[7] Mason G.J., Veasey J.S. (2010). How Should the Psychological Well-being of Zoo Elephants Be Objectively Investigated?. Zoo. Biol..

[8] Mason G., Clubb R., Latham N., Vickery S. (2007). Why and How Should We Use Environmental Enrichment to Tackle Stereotypic Behaviour?. Appl. Anim. Behav. Sci..

[9] Mason G.J., Latham N. (2004). Can’t Stop, Won’t Stop: Is Stereotypy a Reliable Animal Welfare Indicator?. Anim. Welf..

[10] McBride S.D., Long L. (2001). Management of Horses Showing Stereotypic Behaviour, Owner Perception and the Implications for Welfare. Vet. Rec..

[11] Hall N.J. (2017). Persistence and Resistance to Extinction in the Domestic Dog: Basic Research and Applications to Canine Training. Behav. Process..

[12] Carlstead K., Mellen J., Kleiman D.G. (1999). Black Rhinoceros (*Diceros bicornis*) in US Zoos: I. Individual Behavior Profiles and Their Relationship to Breeding Success. Zoo. Biol..

[13] Tuyttens F.A.M., de Graaf S., Heerkens J.L.T., Jacobs L., Nalon E., Ott S., Stadig L., Van Laer E., Ampe B. (2014). Observer Bias in Animal Behaviour Research: Can We Believe What We Score, If We Score What We Believe?. Anim. Behav..

[14] Alghamdi S., Zhao Z., Ha D.S., Morota G., Ha S.S. (2022). Improved Pig Behavior Analysis by Optimizing Window Sizes for Individual Behaviors on Acceleration and Angular Velocity Data. J. Anim. Sci..

[15] Arcidiacono C., Mancino M., Porto S.M.C., Bloch V., Pastell M. (2021). IoT Device-Based Data Acquisition System with on-Board Computation of Variables for Cow Behaviour Recognition. Comput. Electron. Agric..

[16] Turner K.E., Sohel F., Harris I., Ferguson M., Thompson A. (2023). Lambing Event Detection Using Deep Learning from Accelerometer Data. Comput. Electron. Agric..

[17] Hakansson F., Jensen D.B. (2023). Automatic Monitoring and Detection of Tail-Biting Behavior in Groups of Pigs Using Video-Based Deep Learning Methods. Front. Vet. Sci..

[18] Zhang K., Li D., Huang J., Chen Y. (2020). Automated Video Behavior Recognition of Pigs Using Two-Stream Convolutional Networks. Sensors.

[19] Li B., Xu W., Chen T., Cheng J., Shen M. (2023). Recognition of Fine-Grained Sow Nursing Behavior Based on the SlowFast and Hidden Markov Models. Comput. Electron. Agric..

[20] Tu S., Du J., Liang Y., Cao Y., Chen W., Xiao D., Huang Q. (2024). Tracking and Behavior Analysis of Group-Housed Pigs Based on a Multi-Object Tracking Approach. Animals.

[21] Redmon J., Divvala S., Girshick R., Farhadi A. You Only Look Once: Unified, Real-Time Object Detection. Proceedings of the IEEE Conference on Computer Vision and Pattern Recognition.

[22] Gu C., Sun C., Ross D.A., Vondrick C., Pantofaru C., Li Y., Vijayanarasimhan S., Toderici G., Ricco S., Sukthankar R. Ava: A Video Dataset of Spatio-Temporally Localized Atomic Visual Actions. Proceedings of the IEEE Conference on Computer Vision and Pattern Recognition.

[23] Orban D.A., Siegford J.M., Snider R.J. (2016). Effects of Guest Feeding Programs on Captive Giraffe Behavior. Zoo Biol..

[24] Seeber P.A., Ciofolo I., Ganswindt A. (2012). Behavioural Inventory of the Giraffe (*Giraffa camelopardalis*). BMC Res. Notes.

[25] Hara K., Kataoka H., Satoh Y. Learning Spatio-Temporal Features with 3d Residual Networks for Action Recognition. Proceedings of the IEEE International Conference on Computer Vision Workshops.

[26] Feichtenhofer C., Fan H., Malik J., He K. Slowfast Networks for Video Recognition. Proceedings of the IEEE/CVF International Conference on Computer Vision.

[27] Jocher G., Qiu J., Chaurasia A. (2024). Ultralytics Yolo11. *gitHub repository*. https://github.com/ultralytics/ultralytics.

[28] Arnab A., Dehghani M., Heigold G., Sun C., Lučić M., Schmid C. Vivit: A Video Vision Transformer. Proceedings of the IEEE/CVF International Conference on Computer Vision.

[29] Bertasius G., Wang H., Torresani L. (2021). Is Space-Time Attention All You Need for Video Understanding?. arXiv.

[30] Cao J., Pang J., Weng X., Khirodkar R., Kitani K. Observation-Centric Sort: Rethinking Sort for Robust Multi-Object Tracking. Proceedings of the IEEE/CVF Conference on Computer Vision and Pattern Recognition.

[31] Hripcsak G., Rothschild A.S. (2005). Agreement, the f-Measure, and Reliability in Information Retrieval. J. Am. Med. Inform. Assoc..

[32] Sun C., Shrivastava A., Vondrick C., Murphy K., Sukthankar R., Schmid C. Actor-Centric Relation Network. Proceedings of the European Conference on Computer Vision (ECCV).

[33] Tong Z., Song Y., Wang J., Wang L. (2022). Videomae: Masked Autoencoders Are Data-Efficient Learners for Self-Supervised Video Pre-Training. Adv. Neural Inf. Process. Syst..

